# Factors influencing the degree of disability in patients with neuromyelitis optica spectrum disorders

**DOI:** 10.1186/s40001-023-01404-z

**Published:** 2023-10-12

**Authors:** Lili Shi, Dawei Li, Yunxiu Zhang, Jinling Wang, Jingxuan Fu, Xia Liu, Peichang Wang

**Affiliations:** 1 Clinical Laboratory of Xuanwu Hospital, Capital Medical University, Beijing, China; 2 Neurology of Xuanwu Hospital, Capital Medical University, Beijing, China

**Keywords:** Neuromyelitis optica spectrum disorders, Blood–Brain barrier, Antioxidant protein, Degree of disability, EDSS

## Abstract

**Objective:**

To investigate the factors influencing the degree of disability in patients with neuromyelitis optica spectrum disorder (NMOSD) and provide evidence for disease monitoring and clinical intervention.

**Methods:**

Eighty-four patients with NMOSD at Xuanwu Hospital Capital Medical University were enrolled in this retrospective study. Before treatment, blood was collected from all patients, and their expanded disability status scores were assessed.

**Results:**

Of the 84 patients assessed, 66 (78.57%) had an expanded disability status scale score < 7, and 18 (21.43%) had scores ≥ 7. The univariate analysis showed that the total bilirubin (TBil), cerebrospinal fluid albumin (CSF ALB), cerebrospinal fluid immunoglobulin G (CSF IgG), QALB, and QIgG levels in the group with scores ≥ 7 were significantly different from those with scores < 7 (*P* < 0.05). In addition, Spearman’s correlation analysis showed a significant correlation between ALB and expanded disability status scores in patients with NMOSD (*P* < 0.05), and the multivariate logistic regression analysis showed that TBil was an independent factor influencing the degree of disability in patients with NMOSD (*P* < 0.05). The receiver operating characteristic curve was constructed using TBil values; the area under the curve of TBil was 0.729 (*P* < 0.01), and the best cut-off value was 11.015 g/L. Its sensitivity in predicting the severity of disability in NMOSD patients was 51.5% while its specificity was 88.9%.

**Conclusion:**

TBil is an independent factor that influences the severity of disability in patients with NMOSD. In addition, ALB is closely related to NMOSD severity, and some factors associated with the BBB are significantly increased in severely disabled NMOSD patients.

**Supplementary Information:**

The online version contains supplementary material available at 10.1186/s40001-023-01404-z.

## Introduction

Neuromyelitis optica spectrum disorder (NMOSD) is an autoimmune demyelinating disease of the central nervous system characterized by simultaneous or continuous occurrence of optic neuritis and myelitis [1, 2]. NMOSD usually involves severe immune-mediated attacks, resulting in severe disability and high recurrence rates [3, 4]. AQP-4 (AQP4-IgG) and MOG antibody (MOG-IgG) are the most commonly used antibodies for diagnosing NMOSD [5]. Increases in the diagnostic accuracy and awareness NMOSD have led to a calculated NMOSD prevalence rate of 10 per 100,000 people in some geographic areas [6], while more than 100,000 cases have been reported worldwide [7]. NMOSD, especially in patients with severe disabilities, can have a significant negative impact on the quality of life, family, and social relationships of these patients. Therefore, it is important to intensively investigate the factors that influence severe NMOSD disabilities.

Oxidative stress is involved in NMOSD pathogenesis. Oxidative stress refers to an imbalance in reactive oxygen species (ROS) metabolism, which contributes to the pathogenesis of diseases. Immune release of ROS results in demyelination and axonal damage, both of which are exacerbated by weaker cellular antioxidant defense systems and vulnerability to ROS in the central nervous system [8, 9].

The blood–brain barrier (BBB) plays an important role in maintaining the function of the central nervous system. Damage to the BBB allows pathogenic antibodies and inflammatory immune cells from the blood to enter the central nervous system (CNS), which may be part of the pathogenic mechanism of NMOSD. The pathogenesis of NMOSD is closely associated with BBB breakdown and AQP4 antibodies. Many studies have reported that BBB disruption is related to the degree of disability in NMOSD, primarily in the acute phase [4]. However, factors influencing severe disability in NMOSD patients has not been reported. Here, we investigated the significance of several antioxidant proteins and BBB-related markers in patients with NMOSD. We aimed to investigate the factors influencing the severity of disability in NMOSD to provide evidence for early clinical diagnosis, alleviate disease severity, and improve the quality of life of NMOSD patients.

## Materials and methods

### Patients

**Fig. 1:**
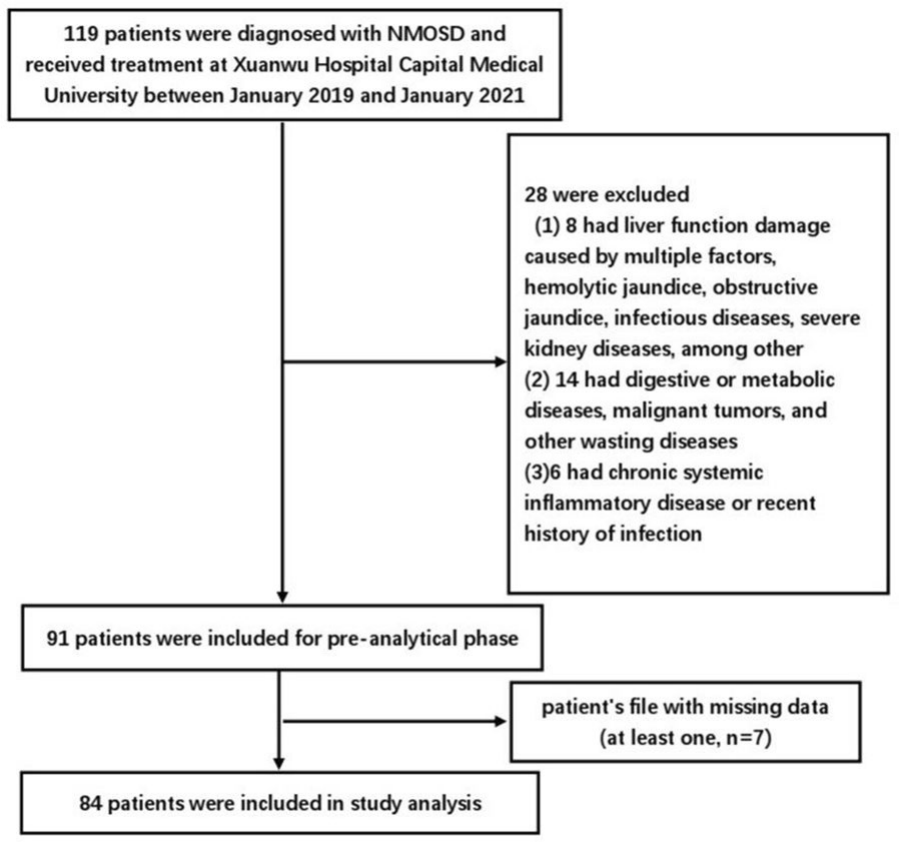
Flowchart

All patients provided informed consent to participate in the study. In this retrospective study, we collected clinical data from patients who were diagnosed with NMOSD and received treatment at Xuanwu Hospital Capital Medical University between January 2019 and January 2021. The inclusion criteria were: (1) diagnosis of NMOSD according to the Wingerchuk standard (2006) or McDonald NMOSD international general diagnostic standard (2015) [10, 11]; and (2) complete clinical data. The exclusion criteria were: (1) liver function damage caused by multiple factors (ALT > 40 g/L, AST > 40 g/L), hemolytic jaundice, obstructive jaundice, infectious diseases, severe kidney diseases; (2) digestive or metabolic diseases, malignant tumors, or other wasting diseases; and (3) chronic systemic inflammatory disease or recent history of infection. This study met the requirements of the World Medical Association Declaration of Helsinki. A total of 84 patients conformed to these criteria and were enrolled in this study (Fig. 1).

### Data collection 

Clinical data were retrospectively obtained using the hospital's electronic record system. The following clinical data were collected: sex, age, total bilirubin (TBil), direct bilirubin (DBil), uric acid (UA), albumin (ALB), immunoglobulin G (IgG), cerebrospinal fluid immunoglobulin G (CSF IgG), cerebrospinal fluid albumin (CSF ALB), and visual impairment status.

Venous whole blood and CSF were collected from all patients who fasted for > 8 h within 24 h of hospitalization. Serum ALB, IgG, CSF IgG, and ALB levels were determined using an IMMAGE 800 Automatic Immunoanalyzer (BECKMAN COULTER, USA). Serum UA, TBil, and DBil levels were determined using a 7600 Series Automatic Analyzer (HITACHI). Calibrating products and quality control products with supporting reagent standards were used, and quality control at high, medium, and low levels was conducted. QIgG (QIgG = IgG (CSF in mg/L)/ IgG (serum in g/L)), QALB (QALB = albumin (CSF in mg/L)/albumin (serum in g/L)), and the 24 h intrathecal synthesis rate were calculated according to the recommendations of Reibers [12].

### Clinical assessment

To determine the extended disability status scale (EDSS) score at admission, at least two professional neurologists carefully reviewed the patients’ clinical records. This was recorded as the initial EDSS score, which was used to assess disease severity. We monitored a milestone in the EDSS assessment of disease disability, namely a severe wheelchair-dependent disability (EDSS ≥ 7) [13–15]. The cohort was divided into mild/moderate disability (EDSS score 0–6.5) and severe disability (EDSS score 7–9.5) groups.

### Statistical analyses

All statistical analysis was conducted in SPSS (version 22) software (SPSS, Inc., Chicago, IL) and GraphPad Prism 8.0 (GraphPad Software, Inc., San Diego, CA, USA). Quantitative data conforming to a normal distribution were represented as mean ± standard deviation [x ± σ], and an inter-group comparison was performed using an independent sample *t*-test. In contrast, quantitative data conforming to non-normal distributions were represented as median and quartile difference [M (P25, P75)], and the Mann–Whitney *U* test was used for the comparison between groups. The correlations between the clinical indices and initial EDSS scores were obtained using Spearmans’ correlation analysis. In addition, qualitative data were compared between the two groups using the chi-squared test. Multiple logistic regression analysis was used to analyze independent risk factors, and stepwise regression was used to establish the model. A receiver operating characteristic curve (ROC) was used to determine the diagnostic ability of ALB and TBil, and the Youden index was used to determine the optimal cut-off value (Youden index = sensitivity + specificity−1). The differences were considered statistically significant at *P* < 0.05.

## Results

### Univariate analysis of the factors influencing the degree of disability in NMOSD patients

The mean age of all patients was 43.13 ± 13.31, and the ratio of men to women was 14/70 (Additional file 1). Among the 84 patients assessed, 66 (78.57%) had an EDSS score < 7, with a mean age of 43.11 ± 13.63 years, including 11 men and 55 women; 18 (21.43%) had an EDSS score ≥ 7, with a mean age of 43.22 ± 12.41 years, including 3 men and 15 women. The univariate analysis showed that TBil, CSF ALB, CSF IgG, QALB, and QIgG in the EDSS score ≥ 7 group were significantly different from those in the EDSS score < 7 group (*P* < 0.05) (Table 1).

**Table 1:** Univariate analysis of the degree of disability in NMOSD patients

Index	< 7 (*n* = 66)	≥ 7 (*n* = 18)	*t*/*z*/*χ*2	*P*
EDSS score	4.50 (4.00, 5.00)	7.00 (6.87, 8.12)	−6.603^c^	0.000^**^
Age	43.11 ± 13.63	43.22 ± 12.41	−0.33^a^	0.731
Men/women ratio	11/55	3/15	0.000^b^	1.000
UA umol/L	249.28 ± 76.53	239.39 ± 88.32	0.470^a^	0.742
TBil umol/L	11.23 (8.09, 14.02)	7.72 (6.64, 10.39)	−2.960^c^	0.003^**^
DBil umol/L	3.27 (2.28, 4.53)	2.58 (2.08, 3.83)	−1.248^c^	0.212
ALB g/L	37.72 ± 3.70	36.35 ± 2.79	1.460^a^	0.856
IgG g/L	10.15 (8.04, 13.72)	10.75 (7.54, 13.95)	−0.349^c^	0.727
CSF ALB mg/dl	17.25 (13.41, 27.07)	22.99 (18.76, 39.94)	−2.333^c^	0.02^*^
CSF IgG mg/dl	2.87 (1.93, 5.14)	6.05 (2.74, 10.65)	−2.398^c^	0.016^**^
QALB	4.7 (3.5, 7.7)	6.3 (5.5, 11.2)	−2.524^c^	0.012^**^
QIgG	2.8 (2.0, 4.5)	4.7 (2.9, 7.1)	−2.660^c^	0.008^**^
24 h intrathecal synthesis rate	3.0 (1.45, 6.96)	5.24 (1.90, 11.52)	−1.157^c^	0.116
Visual impairment	39/27	8/10	1.231^b^	0.267

The univariate analysis showed that TBil, CSF ALB, CSF IgG, QALB, and QIgG levels in the EDSS score ≥ 7 group were significantly different from those in the EDSS score < 7 group (*P* < 0.05)

^*^P < 0.05, ^**^P < 0.01

^a^The t-value from the independent sample t-test

^b^The χ^2^-value from the χ^2^test

^c^The Z-value from the Mann–Whitney U test

### Correlation analysis of factors affecting the disability degree of NMOSD patients

Spearman’s correlation analysis showed that ALB was significantly correlated with the EDSS scores of NMOSD patients (*R* = −0.263, *P* = 0.015) (Fig. 1); however, this correlation was weak. There was no significant correlation between TBil and disease severity (*P* > 0.05) (Table 2).

**Table 2:** Correlation analysis of clinical parameters and initial EDSS scores

Index	*r*	*P*
UA	−0.009	0.936
TBil	−0.189	0.086
DBil	−0.089	0.420
ALB	−0.263	0.015^*^
IgG	−0.049	0.659
CSF ALB	0.064	0.561
CSF IgG	0.115	0.297
QALB	0.105	0.342
QIgG	0.164	0.136
24 h intrathecal synthesis rate	0.130	0.238

^*^P < 0.05; Spearman’s correlation analysis showed that ALB was significantly correlated with the EDSS scores of NMOSD patients (*P* = 0.015)

### Multiple logistic regression analysis of factors influencing the disability degree of NMOSD patients

EDSS scores < 7 and ≥ 7 were set as dependent variables. The indicators TBil, CSF ALB, CSF IgG, QALB, QIgG, and ALB, which were correlated with EDSS in the previous univariate analysis, were used as independent variables in the multivariate logistic regression analysis. The analysis showed that TBil was an independent factor influencing the degree of disability in patients with NMOSD, with statistical significance (*P* < 0.01) (Table 3).

**Table 3:** Multiple logistic regression analysis of factors influencing the degree of disability in NMOSD patients

Index	β	SE	Wald	*P*	OR	OR 95%	
						Lower	Upper
						Limit	Limit
TBil	−0.471	0.152	9.553	0.002^**^	0.624	0.463	0.842
CSF ALB	−0.173	0.267	0.418	0.518	0.841	0.498	1.421
CSF IgG	−0.056	0.111	0.261	0.610	0.945	0.761	1.174
QALB	8.348	9.736	0.735	0.391	4221.618	0.000	8.18E + 11
QIgG	2.329	1.755	1.763	0.184	10.272	0.33	319.969
ALB	0.155	0.255	0.368	0.544	1.168	0.708	1.926

^**^P < 0.01. Multivariate logistic regression analysis showed that TBil was an independent factor influencing the degree of disability in patients with NMOSD (*P* < 0.01)

### Constructing the ROC curve

**Fig. 2:**
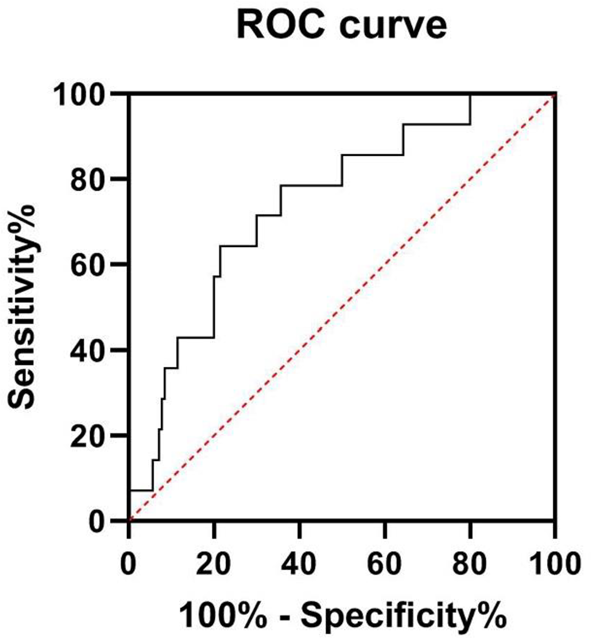
ROC curve of TBil

ALB and TBil levels were used to construct the ROC curve (Table 4). The AUC of TBil was 0.729 (*P* < 0.01), the optimal cut-off value was 11.015 g/L, and the sensitivity and specificity values of TBil for predicting severe disability in NMOSD patients were 51.5% and 88.9%, respectively (Fig. 2).

**Table 4:** ROC analysis results of factors affecting the degree of disability in NMOSD patients

Index	The best cut-off value	AUC (95% CI)	Sensitivity	Specificity	*P*
TBil	11.015	0.729 (0.608, 0.849)	0.515	0.889	0.003^**^
ALB	35.07	0.645 (0.501, 0.789)	0.864	0.500	0.061

^**^P < 0.01. The AUC of TBil was 0.729 (*P* < 0.01), the optimal cut-off value was 11.015 g/L, and the sensitivity and specificity values of TBil for predicting severe disability in NMOSD patients were 51.5% and 88.9%, respectively

## Discussion

Epidemiological studies have shown that the incidence of NMOSD in China is 0.41 per 100,000 people per year. The incidence is higher in women, and the incidence rate ratio (IRR) of women to men is 4.52. This incidence rate increases with age, peaking at 55–64 years in women and 65–74 years in men, and then gradually declines [16]. The degree of disability is an important factor affecting quality of life in patients with NMOSD. Therefore, it is important to explore factors that influence the degree of disability.

Multiple logistic regression analysis showed that TBil level was an independent factor affecting severe disability in patients with NMOSD. Previous studies found that TBil levels in patients with NMO were significantly lower than those in control patients, and the conclusion was the same when men and women were studied separately [17]. However, a relationship between TBil and disease severity has not yet been reported. In our univariate analysis, the TBil concentration in the EDSS score ≥ 7 group was significantly lower than that in the EDSS score < 7 group. Notably, NMOSD is an autoimmune, neurodegenerative, inflammatory disease, and several studies [18–20] have demonstrated a close relationship between bilirubin levels and autoimmune diseases. In one study, serum bilirubin levels of systemic lupus erythematosus patients were found to be significantly lower than those of normal controls [18]. Meanwhile, serum bilirubin levels have been found to decrease more in systemic lupus erythematosus patients than in pleuritic and nephritis patients [19, 20]. In addition, animal experiments have shown that bilirubin effectively prevents the occurrence of experimental autoimmune encephalomyelitis (EAE) and has a positive effect on treatment [21]. In particular, exogenous bilirubin supplementation significantly improved EAE. Therefore, a lack of endocrine-producing bilirubin may significantly exacerbate the course of the disease.

Bilirubin is the final product of the decomposition of heme oxygenase. Many studies had shown that bilirubin is a natural antioxidant and an endogenous scavenger of reactive oxygen species [22, 23]. It inhibits oxidation to a greater extent than several other antioxidants, including alpha-tocopherol, ascorbic acid, and catalase, especially under pathological conditions. Interestingly, just 10 nmol/L of bilirubin protects against nearly 10,000 times the concentration of hydrogen peroxide [24]. In addition, oxidative stress can activate inflammatory cells such as lymphocytes and macrophages, enhance monocyte adhesion and migration across the BBB, and produce a variety of inflammatory mediators, all of which contribute to central nervous system inflammation and demyelinating disease [25]. Moreover, higher antioxidant capacity is associated with reduced disease severity in NMOSD [26], indicating that antioxidant therapy may be an attractive treatment option [24]. Notably, bilirubin has strong immunomodulatory activity [21] as well as neuroprotective properties [27], and increasing evidence suggests that bilirubin has significant therapeutic potential for a variety of diseases [28–30]. Bilirubin may also be significant in the treatment of NMOSD, particularly in the treatment of severe disability and the prevention of disease progression. In this study, it was found that the AUC of TBil was 0.729 (*P* < 0.01) and that the best cut-off value was 11.015 g/L. The sensitivity of TBil for predicting the severity of disability in patients with NMOSD was 51.5%, whereas its specificity was 88.9%. It is, therefore, suggested that when TBil reaches 11.015 g/L, the risk of disability in patients with EDSS ≥ 7 points is greater. This finding may provide a basis for the clinical assessment of disease severity, and close monitoring of TBil levels and early intervention may help improve the severity of the disease in NMOSD patients.

The correlation analysis in this study showed that ALB was negatively correlated with EDSS scores. In a previous study, Yao et al. also reported this correlation [31]. As albumin has both immunomodulatory and anti-inflammatory effects [32], ALB levels are typically reduced during inflammation. Low ALB levels have also been found in some neural autoimmune diseases such as Guillain–Barre syndrome and Myasthenia Gravis. Meanwhile, in inflammatory diseases, cytokines may be important regulators of ALB loss [33]. Conversely, ALB also regulates cytokine expression in an NF-κB-dependent manner [34], playing an active role in many immune and inflammatory diseases [35].

In addition to the functions discussed above, albumin has a direct neuroprotective effect on neurons and glial cells, with studies showing that ALB is beneficial to astrocytes, microglia, and nerve cells [35]. It has also been reported that serum ALB level is an important factor affecting the degree of disability in patients with multiple sclerosis [36]. In addition, ALB has been found to be a predictor of the diagnosis of multiple sclerosis and in distinguishing progressive multiple sclerosis [37]. In their study, Peng et al. [17] found for the first time that ALB levels in NMO patients were lower than those in normal controls and confirmed the importance of ALB in NMO. In another study, Yao et al. [31] reported that ALB was significantly correlated with the EDSS scores of patients with acute NMOSD, which is consistent with our findings. This report also showed that low ALB is an independent influencing factor for severe disability in patients with NMOSD. However, in our study, there was no significant difference in ALB between patients with severe disability and those with less severe disability, and ALB was not found to be an independent influencing factor in the multivariate analysis.

The correlation between ALB levels and disease severity in this study was lower than that reported by Yao et al. The possible reasons for this discrepancy are as follows: 1) According to Yao et al., there is a strong correlation between ALB and NMOSD in the acute stage but no correlation between ALB and NMOSD in the remission stage. However, the number of acute stage patients in their study population was much larger than the number of those in the remission stage. Thus, their data analysis indicated that low ALB was an independent influencing factor of severe disability in NMOSD. (2) The selected EDSS nodes with severe disabilities were inconsistent. Due to these inconsistencies, further studies are required to confirm this hypothesis. Nevertheless, ALB is inextricably linked to the disease mechanism of NMOSD.

Our univariate analysis showed that the CSF ALB, CSF IgG, QALB, and QIgG levels of patients with severe disabilities were significantly higher than those of patients with non-severe disabilities. It has been previously reported [38] that BBB permeability is a biomarker for predicting the severity of NMOSD. EDSS scores and CSF ALB levels in patients with increased BBB permeability were significantly higher than in those with normal BBB permeability, and QALB was positively correlated with the length of myelopathy. In their study, You et al. [39] used QALB levels to evaluate BBB damage and showed that QALB was associated with higher EDSS scores. Although several studies have investigated the relationship between QALB levels and the clinical features of NMOSD [38–40], the effect of QALB on severe disability in NMOSD remains unclear. In this study, significant differences were found among severe and less severe disability degrees and QALB levels, indicating different degrees of BBB dysfunction in patients with severe and less severe disabilities. The data showed that QALB levels were higher and the BBB damage was greater in patients with severe disabilities. CSF IgG and QIgG levels also differed significantly between severe and less severe disability cases. In severe disability patients, the BBB disruption is more profound, and more IgG penetrates the BBB, resulting in increased CSF IgG and QIgG.

The BBB is essential for maintaining the microenvironment of the central nervous system and protecting the central nervous system from peripheral inflammatory factors, bacteria, and viruses. The breakdown of the integrity of the BBB allows immune cells and pro-inflammatory cytokines to enter the central nervous system, ultimately triggering neural inflammatory diseases. Astrocytes are important components of the BBB and may regulate barrier function [41], whereas aquaporin 4 (AQP4) is highly expressed in astrocyte foot processes adjacent to neural vessels, especially in the brain stem, optic spinal cord, and periventricular area [42]. Regions with high AQP4 expression are also typical sites of NMOSD damage [5]. When BBB damage occurs, AQP4 invades the central nervous system and binds to astrocytes, which may trigger an immune cascade that leads to astrocyte death, loss of extracellular and metabolic balance, and further BBB damage [42]. The more severe the BBB damage, the more AQP4 may penetrate the central nervous system, leading to a more severe NMOSD inflammatory process [39]. This also explains the elevations in CSF ALB, CSF IgG, QALB, and QIgG levels in severely disabled NMOSD patients. Therefore, maintaining BBB function is of great significance for the prevention of disease progression to severe disability.

Our study has several limitations. First, we lacked follow-up data for the patients and failed to analyze the relationship between disease progression and prognosis. Second, the study cases could have been divided into acute and remission stages, and the number of cases could have been expanded and evenly distributed in terms of patients in the acute and remission phases.

In conclusion, TBil is an independent factor that influences the severity of disability in NMOSD patients, and ALB is closely related to NMOSD severity. In addition, CSF ALB, CSF IgG, QALB, and QIgG, which are associated with the BBB, are significantly increased in severely disabled NMOSD patients. Close monitoring of associated risk factors and early intervention may help improve the disease severity of NMOSD. Finally, bilirubin has important potential in the treatment of NMOSD, especially in the treatment of severe disability and the prevention of disease progression.

### Supplementary Information

**Additional file 1.** All patients’ demographic data.

## Data Availability

The original data for this article have been uploaded.
