# Supplementary material for: Factors influencing the degree of disability in patients with neuromyelitis optica spectrum disorders
Source: Eur J Med Res. 2023 Oct 12;28:426. doi: 10.1186/s40001-023-01404-z (PMC10568753; doi:10.1186/s40001-023-01404-z)
Supplement: Supplementary file 1 — Additional file 1. All patients’ demographic data. [file 40001_2023_1404_MOESM1_ESM.docx]

**Table**

| **Index** | **all patients** |
| --- | --- |
| EDSS score | 4.50(4.50,6.00) |
| Age | 43.13±13.31 |
| Men/Women ratio | 14/70 |
| UA umol/L | 247.15±78.75 |
| TBil umol/L | 10.41(7.62,13.72) |
| DBil umol/L | 3.18(2.24,4.46) |
| ALB g/L | 37.43±3.56 |
| IgG g/L | 10.20(8.00,13.68) |
| CSF ALB mg/dl | 18.75(14.48,29.68) |
| CSF IgG mg/dl | 3.39(1.96,6.41) |
| QALB | 5.19(3.63,8.1) |
| QIgG | 2.98(2.1,4.9) |
| 24h intrathecal synthesis rate | 3.42(1.54,7.26) |
| visual impairment | 47/37 |
